# Inhibition of Human Malignant Pleural Mesothelioma Growth by Mesenchymal Stromal Cells

**DOI:** 10.3390/cells10061427

**Published:** 2021-06-08

**Authors:** Valentina Coccè, Silvia La Monica, Mara Bonelli, Giulio Alessandri, Roberta Alfieri, Costanza Annamaria Lagrasta, Denise Madeddu, Caterina Frati, Lisa Flammini, Daniela Lisini, Angela Marcianti, Eugenio Parati, Francesca Paino, Aldo Giannì, Giampietro Farronato, Angela Falco, Lorenzo Spaggiari, Francesco Petrella, Augusto Pessina

**Affiliations:** 1 CRC StaMeTec, Department of Biomedical, Surgical and Dental Sciences, University of Milan, 20122 Milan, Italy; valentina.cocce@unimi.it (V.C.); francesca.paino@unimi.it (F.P.); aldo.gianni@unimi.it (A.G.); francesco.petrella@unimi.it (F.P.); augusto.pessina@unimi.it (A.P.); 2 Department of Medicine and Surgery, University of Parma, 43126 Parma, Italy; mara.bonelli@unipr.it (M.B.); roberta.alfieri@unipr.it (R.A.); costanzaannamaria.lagrasta@unipr.it (C.A.L.); denise.madeddu@unipr.it (D.M.); caterina.frati@unipr.it (C.F.); angela.falco@unipr.it (A.F.); 3 Department of Molecular and Translational Medicine, Section of Microbiology and Virology, Medical School, University of Brescia, 25100 Brescia, Italy; cisiamo2@yahoo.com; 4 Food and Drug Department, University of Parma, 43124 Parma, Italy; lisa.flammini@unipr.it; 5 Cell Therapy Production Unit-UPTC, Fondazione IRCCS Istituto Neurologico Carlo Besta, 20133 Milan, Italy; daniela.lisini@istituto-besta.it (D.L.); angela.marcianti@istituto-besta.it (A.M.); 6 IRCCS Istituti Clinici Scientifici Maugeri, 20138 Milan, Italy; eugenio.parati@icsmaugeri.it; 7 Maxillo-Facial and Dental Unit, Fondazione Ca’ Granda IRCCS Ospedale Maggiore Policlinico, 20122 Milan, Italy; giampietro.farronato@unimi.it; 8 Unit of Orthodontics and Paediatric Dentistry, Fondazione Ca’ Granda IRCCS Ospedale Maggiore Policlinico, 20122 Milan, Italy; 9 Department of Thoracic Surgery, IRCCS European Institute of Oncology, 20139 Milan, Italy; lorenzo.spaggiari@ieo.it; 10 Department of Oncology and Hemato-Oncology, University of Milan, 20122 Milan, Italy

**Keywords:** mesenchymal stromal cells, mesothelioma, malignant pleural mesothelioma (MPM), cell therapy

## Abstract

Background: Malignant Pleural Mesothelioma (MPM) is an aggressive tumor that has a significant incidence related to asbestos exposure with no effective therapy and poor prognosis. The role of mesenchymal stromal cells (MSCs) in cancer is controversial due to their opposite effects on tumor growth and in particular, only a few data are reported on MSCs and MPM. Methods: We investigated the in vitro efficacy of adipose tissue-derived MSCs, their lysates and secretome against different MPM cell lines. After large-scale production of MSCs in a bioreactor, their efficacy was also evaluated on a human MPM xenograft in mice. Results: MSCs, their lysate and secretome inhibited MPM cell proliferation in vitro with S or G0/G1 arrest of the cell cycle, respectively. MSC lysate induced cell death by apoptosis. The efficacy of MSC was confirmed in vivo by a significant inhibition of tumor growth, similar to that produced by systemic administration of paclitaxel. Interestingly, no tumor progression was observed after the last MSC treatment, while tumors started to grow again after stopping chemotherapeutic treatment. Conclusions: These data demonstrated for the first time that MSCs, both through paracrine and cell-to-cell interaction mechanisms, induced a significant inhibition of human mesothelioma growth. Since the prognosis for MPM patients is poor and the options of care are limited to chemotherapy, MSCs could provide a potential new therapeutic approach for this malignancy.

## 1. Introduction

Malignant Pleural Mesothelioma (MPM) is an aggressive tumor that has a significant incidence related to widespread asbestos exposure [1]. There is still no effective therapy for MPM patients, the prognosis is poor and, furthermore, conventional chemotherapy entails remarkable toxic side effects without a clear clinical benefit [2,3,4,5].

As discussed in many reviews, the relationships between cancer and mesenchymal stromal cells (MSCs) are a controversial matter because MSCs can exert opposite effects on tumor growth. A tumor-promoting capacity of MSCs has been described as the result of paracrine activity of growth factors, exosomes and anti-apoptotic molecules secreted by MSCs [6,7]. Furthermore, the ability of MSCs to differentiate into tumor-associated fibroblasts has been suggested to be a mechanism able to stimulate tumor growth, metastasis formation and increase drug-resistance [8].

On the other hand, many reports suggest that MSCs and/or MSCs-derived secretome can exert antitumor activity against many different types of cancers such as leukemia, prostate carcinoma, colon carcinoma, and breast cancer, both in vitro and in vivo models [9,10,11,12]. To explain these anticancer effects, different mechanisms have been investigated such as apoptosis induction due to Tumor Necrosis Factor-Related Apoptosis-Inducing Ligand (TRAIL) up-regulation, cell cycle arrest, over-expression of tumor suppressor genes, and/or cytokine-mediated process [13]. By homing to the tumor site, MSCs can integrate into the tumor mass and exert suppression of growth, as reported for SKMES1 and A549 lung adenocarcinoma cells via the production of some soluble factors [14] or other mechanisms [15]. In addition to the abovementioned mechanisms, MSCs can impact on tumor growth and progression by modulating the innate and adaptive immune response [16]. All the controversial different roles of MSCs have been discussed in many reviews [17,18] and some authors consider that the discrepancies in the ability of MSCs to promote or suppress cancer could be attributable to many factors such as: the remarkable differences among tumor models, the MSCs tissue source, the amount of MSCs used or the mode of cell administration, and sometimes, the different criteria to select the experimental controls.

In the literature, there are only a few reports regarding the role of MSCs in MPM. A significant tumor-inhibiting effect in vitro on MPM cell lines exerted by conditioned medium from human lung MSCs was reported [19] and a reduction in tumor growth in an in vivo MPM model after intravenous delivery of TRAIL-expressing MSCs has been described [20,21]. In this context, the aim of this study was to investigate the in vitro efficacy of human adipose tissue-derived MSCs (AT-MSCs), their cell lysates and secretome on the proliferation of three human mesothelioma cell lines. A further large-scale bioreactor production of MSCs has been set up for treating the human mesothelioma mouse xenograft model. Our in vitro results demonstrated that MSCs, lysates and conditioned media (CM) from AT-MSCs inhibited the proliferation of the mesothelioma cells. The in vivo study confirmed that a loco-regional treatment of a well-established mesothelioma xenograft with AT-MSCs resulted in a substantial inhibition of tumor growth that was comparable with that produced by the chemotherapeutic drug Paclitaxel (PTX), given through a systemic administration.

## 2. Materials and Methods

### 2.1. Tumor Cell Lines

The human MPM cell lines MSTO-211H (biphasic histotype), NCI-H2452 (epithelioid histotype) and NCI-H2052 (sarcomatoid histotype) were obtained from ATCC (Manassas, VA, USA), which authenticates the phenotypes of these cell lines on a regular basis. Cells were cultured in RPMI-1640 supplemented with 10% Fetal Bovine Serum (FBS, Euroclone, Milan, Italy) and maintained at 37 °C in a water-saturated atmosphere of 5% CO_2_ in air. The cell lines were routinely tested for mycoplasma contamination.

### 2.2. Mesenchymal Stromal Cells (MSCs) Expansion and Characterization

Adipose tissue lipoaspirates were collected, under general anesthesia, from healthy volunteer donors undergoing plastic surgery for aesthetic purposes (age ranged from 18 to 66 years). Samples were collected after signed informed consent of no objection for the use for research of surgical tissues (otherwise eliminated) in accordance with the Declaration of Helsinki. Informed consent was obtained prior to tissue collection; the Institutional Review Board of the IRCCS Neurological Institute C. Besta Foundation approved the design of the study.

MSC starting batches (MSCs expanded in flasks up to passage 3) were characterized analyzing: (a) the typical spindle-shaped MSC morphology and the adhesion capacity to the plastic support assessed at every culture passage of each MSC line; (b) MSCs’ viability assessed at every culture passage of each MSC line (cut-off value ≥ 75%); (c) the percentage of expression of typical MSC markers CD90, CD105 and CD73 (cut-off value ≥ 80%) and the absence of the hematopoietic/endothelial markers CD34, CD45 and CD31 (cut-off value ≤ 15%) by flow cytometry analysis on each culture at passage 3; (d) population doubling time at every culture passage of each line. The osteogenic, adipogenic and chondrogenic differentiation capacities of MSCs were evaluated as previously reported [22]. After expansion in the bioreactor, MSCs were counted and frozen. An aliquot of cells was dedicated to the following controls: (a) viability; (b) flow cytometry analysis (same markers and cut-off as the MSCs starting batch).

### 2.3. MSCs for In Vitro Studies

For in vitro studies, adipose tissue-derived mesenchymal stromal cells (AT-MSCs) were expanded in 25 cm^2^ flasks at a density of 1.2 × 10^4^ cells/cm^2^ in 5mL of DMEM low glucose medium (Euroclone) supplemented with 5% platelet lysate Stemulate (Cook Regentec, Indianapolis, IN, USA) and 2 mM L-glutamine (Euroclone) and incubated at 37 °C, 5% CO_2_. To study the MSCs’ secretome, the conditioned medium of the cells (MSCs CM) obtained from three different donors was collected after 6 days of culture and stored in 1 mL aliquots at −80 °C. To study the cell lysate (MSCs LYS), the cell monolayers were detached with trypsin-EDTA (Euroclone) and after counting, the cells were suspended in 3 mL of complete medium and lysed through a sonication procedure (Labsonic UBraun, Reichertshausen, Germany). The procedure was performed by three cycles of 0.4 s pulse at 30% amplitude each; then, the sample was centrifuged 10 min at 2500× *g* to eliminate debris and the supernatant (MSCs LYS) was stored at −80 °C until use.

### 2.4. Large Scale Expansion of MSCs

Starting from the lipoaspirate of a donor, the AT-MSCs were expanded in flasks until a number of at least 20 × 10^6^ cells was reached for each passage, not exceeding P3. In order to produce a high amount of cells for in vivo experiments, the MSCs were expanded using the bioreactor Quantum Cell Expansion System (Terumo BCT Inc., Lakewood, CO, USA) and GMP-compliant reagents, as previously described [23]. Briefly, after priming of the disposable expansion set (the bioreactor was coated overnight with 5 mg of human fibronectin (Corning Incorporated, Deeside, UK)) to promote cell adhesion, a 4 L media bag was then attached to the appropriate inlet line on the Quantum disposable expansion set. The expanded MSCs were analyzed for the expression of the typical MSCs markers (CD90, CD73, CD105) by using monoclonal antibodies (Becton Dickinson, Franklin Lake, NJ, USA), as previously described [22].

### 2.5. Analysis of Cell Proliferation, Cell Death and Cell Cycle

Both MSCs LYS and MSCs CM were tested in vitro for their anti-proliferative activity on MSTO-211H, NCI-H2452 and NCI-H2052 cells in 96 multi-well plates (Sarstedt, Nümbrecht, Germany), as previously described [23,24]. Briefly, 1:2 serial dilutions MSCs LYS and MSCs CM were performed in 100 µL of culture medium/well, and then, 10^3^ tumor cells were added to each well. Cell growth was evaluated after 7 days of culture by measuring the optical density at 550 nm in an MTT (3-(4,5-dimethyl-2-thiazolyl)-2,5- diphenyl-2-H-tetrazoliumbromide) assay [25]. Cell death was assessed by Hoechst 33342 and propidium iodide dual staining and by using the Apoptosis/Necrosis Detection Kit (Abcam, Cambridge, UK). Caspase-3 activity was measured by the Caspase-3 Assay Kit (Abcam) following the supplier’s protocols. The distribution of the cells in the cell cycle (determined by PI staining and flow cytometry analysis) was determined as described elsewhere [26].

### 2.6. Transwell Assay

The effect of MSCs on MSTO-211H, NCI-H2452 and NCI-H2052 cell proliferation was analyzed using transwell inserts. Aliquots (2 × 10^4^; 4 × 10^4^; 8 × 10^4^) of MSCs were seeded in a 24-well plate (diameter 1.9 cm^2^), while 1 × 10^3^ MSTO-211H or NCI-H2452 or NCI-H2052 cells were seeded (ratio 1:6; 1:13; 1:26) onto the insert (0.4 µm pore size; Becton Dickinson). After 5 days of incubation (37 °C, 5% CO_2_), the cells in the insert were stained with 0.25% crystal violet (Sigma Aldrich, St. Louis, MO, USA) for 10 min, washed with PBS buffer and eluted with 0.3 mL of 33% glacial acetic acid. The absorbance of the eluted dye was measured at 550 nm.

### 2.7. Cytokines Measure in MSCs Secretome

The conditioned media of standardized cultures of MSCs (1.2 × 10^4^ cells/cm^2^ at 6 days of culture) were analyzed for cytokine content. The qualitative/quantitative analysis was performed by using “multiplex bead-based xMAP technology” (Bio-Plex Human Cytokine 27-Plex Panel, Bio-Plex Human Group II Cytokine 21-Plex Panel, Bio-Rad Laboratories (Hercules, CA, USA)).

### 2.8. Direct Tumor MSCs Cells Interaction

To study the direct interaction between mesothelioma and mesenchymal stromal cells, MSTO-211H cells were co-cultured with fluorescent MSCs (hASCs-TS/GFP^+^) that were previously established in our laboratory [27,28,29]. The study was performed by using a cyto-inclusion technique and the relationship between MSCs and tumor was analyzed under a confocal microscope [30]. Briefly, MSTO-211H cells (5 × 10^6^) were co-cultured with hASCs-TS/GFP^+^ (at ratio 5:1 tumor/MSCs) and after 72 h of incubation, the mixed cell cultures were detached by trypsin and centrifuged. The final pellets were then gently resuspended in 40 µL of Matrigel (Corning, NY, USA) and, after gelification for one hour at 37 °C, were fixed in paraformaldehyde 4% for 15 min at room temperature. Cyto-included were placed onto slides permeabilized with 0.2% TritonX-100 for 5 min at room temperature, washed once in PBS and stained with 1 ug/mL DAPI (PBS) for 3 min to be observed under a confocal microscope. To confirm the direct activity of hASCs-TS/GFP^+^ against MSTO-211H, specific co-cultures were set up by seeding 5 × 10^3^/cm^2^ of MSTO-211H cells in 24 well plates in the presence of a different ratio (1:1 and 1:2) of hASCs-TS/GFP^+^. The co-cultures were incubated for 48 h under standard culture conditions, then the cells were detached (0.25% trypsin in 0.2 mM EDTA) and counted under fluorescence microscopy.

### 2.9. In Vivo Experiments

A total of 10^6^ human MPM MSTO-211H cells were suspended in 200 μL of Matrigel (Corning) and PBS (1:1) and were subcutaneously injected in the right flank of 6-week-old Balb/c-Nude female mice (Charles River Laboratories, Calco, Italy). The animals were housed in a protected unit for immunodeficient animals with 12 h light–dark cycles and provided with sterilized food and water ad libitum. When tumor volume reached an average size of 100 mm^3^, the animals were randomized into three groups: control (CTRL, n = 7), paclitaxel (PTX, n = 8) and mesenchymal cells (MSCs, n = 7). Once a week, paclitaxel (20mg/kg) or vehicle alone (control group) was administered intraperitoneally and a total of 5 × 10^6^ of MSCs in 200 μL of Matrigel and PBS (1:1) were subcutaneously injected very close to the tumor. The treatments were repeated four times, at day 0, 7, 14 and 21 and then, suspended for a further two weeks, to evaluate tumor mass growth after stopping treatments. Tumor xenografts were measured three times per week using a digital caliper and tumor volume was determined using the formula: (length × width^2^)/2 as previously described [31]. At the same time, animal body weight, posture and gait were monitored. At day 35, mice were euthanized by cervical dislocation and the tumor nodules were collected for further analyses. All experiments involving animals and their care were performed with the approval of the Local Ethical Committee of University of Parma (Organismo per la Protezione e il Benessere degli Animali, OPBA) and of the Italian Ministry of Health, in accordance with the institutional guidelines that are in compliance with national (D.Lgs. 26/2014) and international (Directive 2010/63/EU) laws and policies.

### 2.10. Histological and Morphometric Analysis of Tumor Xenografts

Subcutaneous nodules were excised, formalin fixed, paraffin embedded and processed for histochemical analysis. The morphometric evaluation of xenograft composition was performed on Masson’s trichrome-stained sections. In detail, the number of points overlying neoplastic tissue, fibrosis or necrosis was counted and expressed as percentage of the total number of points explored to define the volume fractions of each tissue component. All these morphometric measurements were obtained with the aid of a grid, defining a tissue area of 0.22 mm^2^ and containing 42 sampling points, each covering an area of 0.0052 mm^2^. These evaluations were performed on the entire section of each tumor sample using an optical microscope (200× final magnification) [32]. To test angiogenesis in the tumor xenografts, some histological sections were processed for immunohistochemistry after antigen retrieval pretreatment (Proteinase K Working Solution, 20 μg/mL for 10–20 min at 37 °C in humidified chamber). Detection of CD31 antigen was performed using primary anti-CD31 antibody (CD31/PECAM-1 (H-3): sc-376764 Santa Cruz, Dallas TX) and IHC Select^®^ Immunoperoxidase Secondary Detection System (Millipore, Burlington, MA, USA) following the manufacturer’s procedures.

### 2.11. Statistical Analysis

Data are expressed as the mean ± standard error (SEM). For statistics, one-way analysis of variance (ANOVA), linear regression analysis and Student’s t-test were performed by using GraphPad Prism 6.0 (GraphPad Software, La Jolla, CA, USA). For in vivo studies, comparison among groups was made using two-way repeated measures ANOVA followed by Bonferroni’s post hoc test (to adjust for multiple comparisons) and/or Student–Newman–Keuls Multiple Comparisons Test. *p* values of less than 0.05 were considered statistically significant.

## 3. Results

### 3.1. Effects of MSCs Lysate and Secretome on Proliferation of Mesothelioma Cell Lines

**Figure 1:**
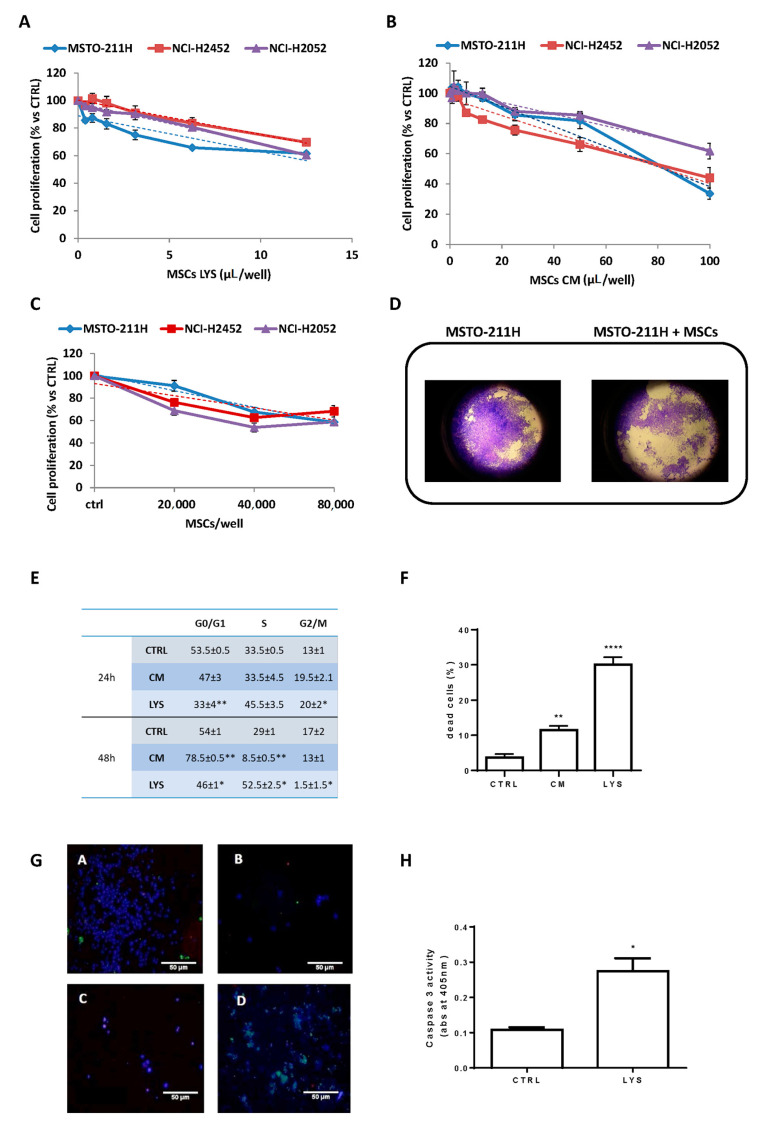
Inhibitory effect of MSCs LYS and MSCs CM on mesothelioma proliferation and co-culture assay. The activity on cell proliferation of MSCs LYS (**A**) and MSCs CM (**B**), expressed as µL/well from 3 donors of adipose tissue-derived MSCs, were evaluated after 7 days in MSTO-211H, NCI-H2452 and NCI-H2052 cell lines by an MTT assay. Each point represents the mean ± standard error (SEM) of three replicates. Linear regression was reported (dashed lines). (**C**) MSTO-211H, NCI-H2452 and NCI-H2052 cells were co-cultured with MSCs and after 5 days, cell proliferation was evaluated by a crystal violet assay. Data are means ± SEM of six independent replicates. Linear regression was reported (dashed line). (**D**) Representative images of transwell inserts with MSTO-211H cells co-cultured alone or in the presence of 8 × 10^4^ MSCs after staining with crystal violet (400×). (**E**) MSTO-211H cells were treated with MSCs LYS (1:4) and MSCs CM (1:2). After 24 and 48 h, cells were stained with propidium iodide and analyzed by flow cytometry for cell cycle phase distribution. Percentage values ± SEM of two independent experiments are reported in the table (* *p* < 0.05, ** *p* < 0.01 vs. control). (**F**) MSTO-211H cells were treated with MSCs LYS (1:4) and MSCs CM (1:2). After 72 h, cell death was quantified by fluorescence microscopy analysis on Hoechst 33342 and propidium iodide-stained cells. Data are expressed as percentage values ± SEM of three independent experiments (** *p* < 0.01, **** *p* < 0.0001 vs. control). (**G**) Representative confocal images of control cells (**A**,**C**) and cells treated for 48 h with MSCs LYS 1:2 (**B,D**). Healthy viable cells were stained with CytoCalcein Violet 450 (blue), necrotic cells with 7-aminoactinomycin D (red), and apoptotic cells with phosphatidylserine (green). (**A**,**B**): adherent cells; (**C**,**D**): detached cells. (Objective 100×). (**H**) Caspase-3 activity measurement after 48 h of treatment. The histogram represents absorbance (abs) at 405 nm. Data are expressed as means ± SEM of three replicates. * *p*<0.05 vs. control.

The lysates (MSCs LYS) and the conditioned medium (MSCs CM) of MSCs obtained from three different donors have been tested concerning the proliferation of MSTO-211H, NCI-H2452 and NCI-H2052 mesothelioma cell lines. Both the lysates and the conditioned medium produced a significant inhibition of the proliferation of mesothelioma cell lines (Figure 1A,B). The dose–response kinetics showed a significant slope (*p* < 0.05) of linear regression with high correlation coefficients (R2 ranged from 0.77 to 0.97). These results demonstrated the paracrine action of MSCs acting without cell-to-cell contact and were also confirmed in a co-culture transwell system. Indeed, as shown in Figure 1C, MCSs produced factors which inhibited mesothelioma cells proliferation and this effect was correlated with the amount of MSCs seeded. The picture of Figure 1D shows representative images of the monolayer of control MSTO-211H in comparison to MSTO-211H cells co-cultured in transwell with 8 × 10^4^ MSCs. We then performed cell cycle analysis and a deeper evaluation of cell viability and cell death in MSTO-211H cells. After 24 h of exposure to MSCs LYS, a block in the S phase of the cell cycle was observed, while the exposure to MSCs CM induced a G0/G1 arrest after 48 h (Figure 1E). Cell death was present in both conditions (Figure 1F); however, apoptosis was documented only with MSCs LYS, as confirmed by morphological analysis (Figure 1G) and caspase 3 activation (Figure 1H).

### 3.2. Direct Effect of MSCs on Proliferation of Mesothelioma Cells

**Figure 2:**
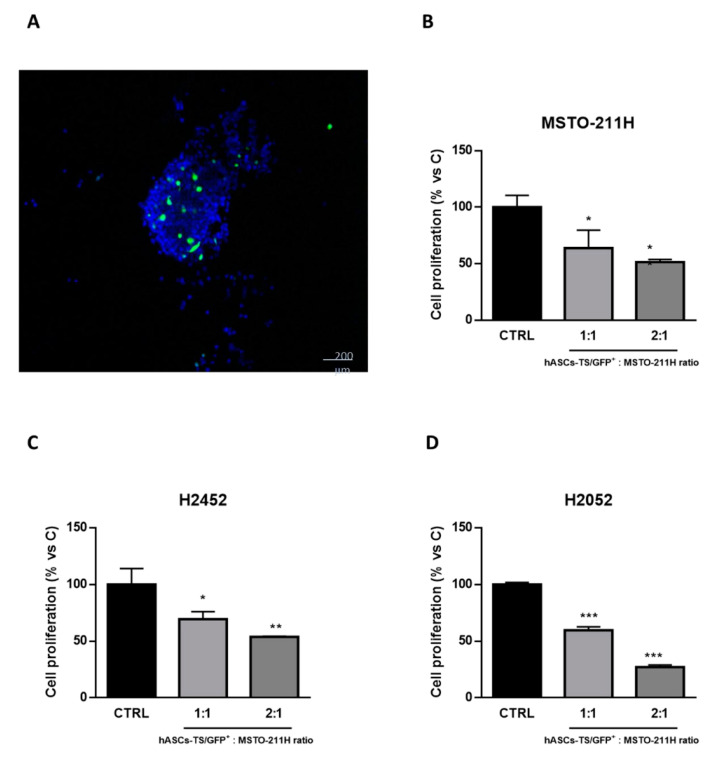
Interaction of MSCs with MSTO-211H cells in vitro. (**A**) The cell-to-cell interference/interaction between MSCs and tumor cells was evaluated by mixing fluorescent MSCs (hASCs-TS/GFP^+^) with MSTO-211H cells and further analyzing the cyto-inclusion under a confocal microscope. (**B**) MSTO-211H, NCI-H2452 and NCI-H2052 cells were co-cultured in absence (ctrl) or in presence of hASCs-TS/GFP^+^ cells in the ratio of 1:1 or 1:2. After 2 days for MSTO-211H and after 4 days for NCI-H2452 and NCI-H2052, the cells were detached and counted under a fluorescence microscopy. Data are the means ± SEM of 3 replicates. * *p* < 0.05, ** *p* < 0.01, *** *p* < 0.001 vs. control.

The cell-to-cell interference/interaction between MSCs and tumor cells was studied by mixing fluorescent MSCs (hASCs-TS/GFP^+^) with MSTO-211H cells and further analyzing the cyto-inclusion under a confocal microscope (Figure 2A). Both MSTO-211H and hASCs-TS/GFP^+^ cells are present in the cell mass according to the initial seeding ratio and hASCs-TS/GFP^+^ cells do not appear to be affected by the interaction with cancer cells.

Specific co-culture experiments showed that the proliferation of MSTO-211H, NCI-H2452 and NCI-H2052 cells was significantly impaired by the interaction with hASCs-TS/GFP^+^ cells, and this anti-proliferative effect was more pronounced in the presence of the higher ratio of hASCs-TS/GFP^+^: cancer cells (Figure 2B–D).

### 3.3. Cytokines Analysis of Secretome

**Figure 3:**
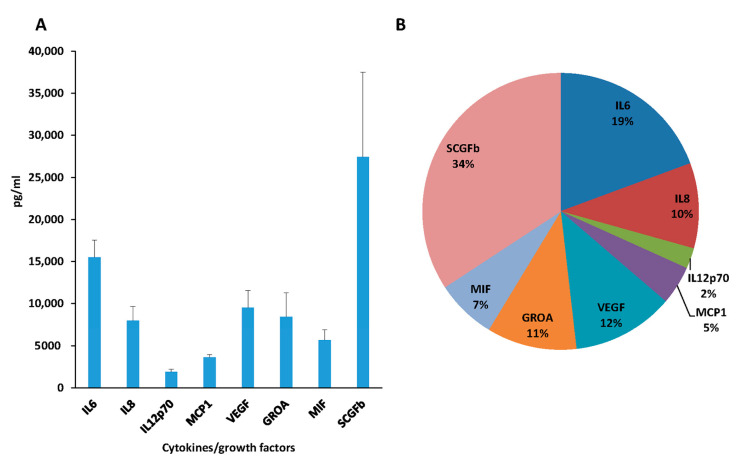
MSCs secretome analysis. (**A**) The histogram reports eight cytokines/growth factors measured in MSC-conditioned media (expressed in pg/mL). Each point represents the mean ± standard error (SEM) of the determinations performed on nine different MSCs donors. (**B**) Cytokines/growth factors expressed as percentage calculated on the total amount of the eight molecules.

The secretome analysis of the MSCs derived from nine different donors was performed on the conditioned/culture medium, by a qualitative/quantitative measure of 38 cytokines. Based on the quantitative analysis of cytokines/growth factors secreted, only eight molecules were produced over 2000 pg/mL. As shown in Figure 3, even if an individual variability was detected, a statistically significant difference in cytokines/growth factors production (*p* < 0.02) was found, with the lowest level for IL12p70 (1925.04 ± 298 pg/mL, 2% of the total amount) and the highest release for SCGFb (27.419 ± 10.073 pg/mL, 34% of the total amount).

### 3.4. Large-Scale Expansion

**Figure 4:**
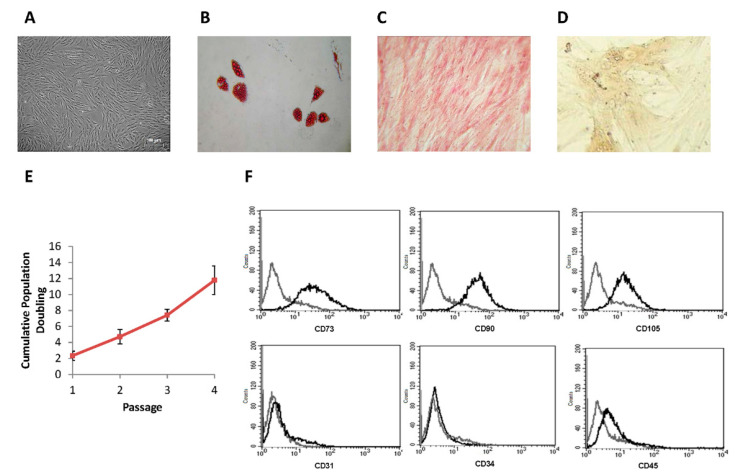
MSC characterization. (**A**): Spindle-shaped MSCs morphology (magnification 5×). (**B**): Adipogenic differentiation evaluated by Oil Red staining (presence of red cytoplasmic inclusions); (**C**): Osteogenic differentiation evaluated by Alizarin Red S staining; (**D**): Chondrogenic differentiation evaluated by micro-mass methods. (All at 200× magnification). (**E**): Number of cumulative population doubling with respect to cellular passage. Each point represents the mean ± SEM of 14 replicates. (**F**): Phenotypic characterization by FACS of a representative sample of MSCs.

The MSCs were expanded in a closed bioreactor, and after 6 days, we obtained 480 × 10^6^ MSCs with a viability of 94%. All the cultured cells obtained displayed the typical MSCs spindle-shaped morphology and a high adhesion capacity to the plastic support (Figure 4A). The MSCs obtained after the expansion also displayed differentiation ability into adipogenic, osteogenic and chondrogenic elements, as reported in Figure 4B–D, respectively. The amount of cumulative population doubling is shown in Figure 4E and it was similar to that of original MSCs. Population doubling time values were of 35.34 ± 9.07 h at P2 and 38.51 ± 11.82 h at P3. Flow cytometry analysis confirmed that MSCs after bioreactor expansion displayed a pattern of CD expression typical of MSCs being CD90^+^, CD105^+^ and CD73^+^ and negative for CD31, CD34 and CD45. In addition, the profile of cytokine production was in the range of the above-described production (Figure 4F). Aliquots of cells, frozen to be used in in vivo experiments, showed a good performance with a high recovery and viability confirmed after 6 months of storing in liquid nitrogen [23].

### 3.5. In Vivo Efficacy of MSCs

**Figure 5:**
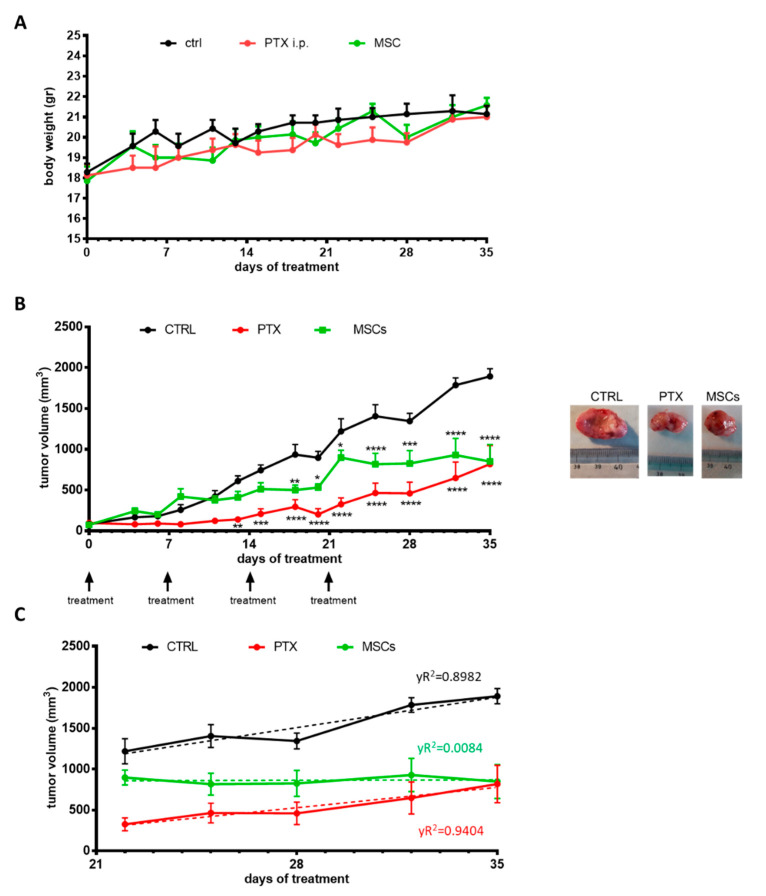
Effects of MSCs in MSTO-211H xenograft model. (**A**) MSTO-211H cells were subcutaneously inoculated into BALB/C nude mice, and after tumors had reached an average size of approximately 100 mm^3^, the animals were treated once a week (at days 0, 7, 14 and 21) with vehicle alone (CTRL), paclitaxel (20 mg/kg) or MSCs (5 × 10^6^). (**A**) Mice body weight was monitored for the entire duration of the treatment. (**B**) Tumor volumes were measured twice per week and data are expressed as means of ± SEM. * *p* < 0.05, ** *p* < 0.01, *** *p* < 0.001, **** *p* < 0.0001 vs. CTRL. Representative images of dissected xenograft tumors are shown. (**C**) The graph shows the tumor growth during the 14 days after stopping treatments (from 22 to 35 days). Data are expressed as means ± SEM and for each group, the linear regression (dashed line) with R2 value is reported.

The efficacy of mesenchymal stromal cells on tumor growth was investigated on MSTO-211H xenograft models. MSTO-211H cells were subcutaneously inoculated into Balb/c-Nude female mice and after tumors had reached an average size of about 100 mm^3^, the animals were randomized into three different groups: control (CTRL), paclitaxel i.p. (PTX) and MSCs. Tumor growth was monitored for 35 days and during this period, the mice showed no signs of toxicity and regularly gained body weight (Figure 5A); no animal death was observed. As illustrated in Figure 5B, the systemic treatment with PTX produced a reduction in tumor growth kinetics, and 14 days after the last treatment (on day 35), the mean tumor volume was of 817 ± 228 mm^3^, which was significantly lower if compared with the mean tumor volume of untreated mice (1893 ± 93 mm^3^; *p* < 0.0001). Moreover, the local administration of MSCs showed a significant reduction in tumor volume which was similar to that of mice receiving the systemic administration of PTX. By comparing the mean tumor volumes at day 22 (Figure 5C), it seems that systemic chemotherapy exerted more efficacy than MSCs in controlling the tumor growth (325 ± 78 mm^3^ versus 898 ± 90 mm^3^; *p* < 0.001). However, after stopping the therapy, in mice treated with PTX the tumors restarted to growth with a significant increase in the mean tumor volume (from 325 ± 78 mm^3^ to 817± 228 mm^3^; *p* < 0.05) underlined also by the significant R2 (0.94). By contrast, no relapses were observed in mice treated with MSCs (Figure 5C).

### 3.6. Histology and Morphometric Analysis of Xenograft

**Figure 6:**
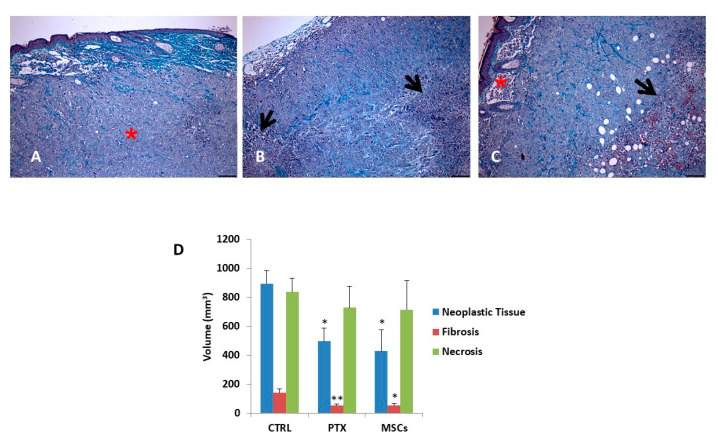
Morphometric analysis of tumor xenografts. Selected sections from MSTO-211H xenografts untreated (CTRL, **A**), treated with paclitaxel (PTX) (**B**) or with MSCs (**C**) stained by Masson’s Trichrome to distinguish the fibrotic tissue (greenish) from neoplastic cells (purple). Black arrows in B and C point to necrotic areas, some of which show pigmented (reddish) debris. Red asterisks in A and C indicate skin and adnexa. Scale bars = 100 µm. (**D**) Bar graph showing the quantitative evaluation of tissue composition (neoplastic tissue, fibrosis and necrosis) in tumor xenografts. Data are expressed as means ± SEM. * *p* < 0.05, ** *p* < 0.02 vs. control group.

The morphometric evaluation allowed to quantify the percentage of tissue occupied by the neoplastic, fibrotic and necrotic component (Figure 6A–C) on Masson’s Trichrome-stained sections of subcutaneous MSTO-211H tumor nodules from each experi-mental group. As shown in Figure 6D, the volume of each component was calculated on the whole masses explanted at day 35 and the volume referred to the neoplastic tissue in untreated mice was compared to that of mice treated with PTX or MSCs. Data analysis by the Student–Newman–Keuls Multiple Comparisons Test indicated a significant decrease (*p* < 0.02) in the fraction of tissue occupied by tumor cells, following administration of PTX i.p. or MSCs. On the contrary, there were no relevant variations in the percentage of necrotic or fibrotic tissues that represent the main tissue component. To test whether angiogenesis in the tumor was affected by MSCs, we evaluated the expression of CD31 in the explanted tumors nodules and we observed that the staining pattern in MSTO-211H treated with MSCs was similar to that of the untreated MSTO-211H (Figures S1 and S2).

## 4. Discussion

The role of MSCs in neoplastic growth is controversial due to their pleiotropic activity and a recent systematic review summarized the application of MSCs of human origin in experimental anticancer therapies [33]. Few data have been reported in the literature on MSCs’ role in mesothelioma and these observations regard in vitro studies on the anticancer activity of the secretome of lung-derived human MSCs [19]. Our preliminary experiments investigated if even the secretome from MSCs expanded from human adipose tissue (easier to isolate respect to MSCs from lung tissue) had some inhibitory activity against three MPM cell lines (MSTO-211H, NCI-H2452 and NCI-H2052). We found that both the cell lysates and the MSC-conditioned medium produced a significant inhibition of MPM cell proliferation with cell cycle arrest. In addition, MSC lysate induced apoptosis. The analysis of 38 molecules detected in the secretome of MSCs from nine different donors indicated a strong difference in their relative amount as also previously reported by other authors [34,35,36]. Even by only considering the molecules produced over 2000 pg/mL, our results did not allow us to reach a clear-cut conclusion to attribute the observed inhibition of mesothelioma cell growth to recognized anticancer molecules secreted by MSCs. In fact, the panel of the factors secreted at the highest level contains molecules that have been described as capable of expressing different and sometimes contradictory/discrepant activities. In this context, even the production of TRAIL (which has been reported as a possible anticancer molecule) was detectable only in two out of nine donor samples at a level (569.3 ± 325 pg/mL) that does not seem sufficient to demonstrate a significant anticancer activity. Regarding interleukin 6 (IL6), it has been reported that this cytokine is present in high concentration in the sera of patients with different cancers including mesothelioma. However, in mesothelioma, the high serum level of IL6 has been indicated as a poor prognostic factor [37]. Many other inflammatory mediators such as chemokines or growth factors produced by MSCs may exert different actions inside the tumor microenvironment involving both antitumor and pro-tumor activity [38]. The conditioned medium of MSCs contains an enormous quantity of molecules (cytokines, chemokines, hormones and many other factors) with different biological activity that can also work together with exosomes/microvesicles produced by MSCs [39,40]. As reported by Mirabdollahi et al. [41], the secretome of umbilical cord-derived mesenchymal cells can have an anticancer effect on MCF-7 tumor cells by inducing apoptosis in a dose-dependent manner. Therefore, although our preliminary study confirmed the anti-proliferative activity of MSCs secretome, it did not help to identify one or more molecules with recognized antitumor activity, suggesting that the observed anticancer activity could be the result of more factors acting together. Moreover, the secretome may change over time when MSCs interact with cancer cells and the secretome of naïve MSCs may be different after co-culturing MSCs with mesothelioma cancer cells. Of course, it is also important to take into account the limitation of in vitro studies performed with MSC secretome because MSCs are physiologically well integrated into very different tissues and their functions are strongly related to the type of tissue in which they are working and to cell-to-cell interaction [42]. In our in vivo model, the cytokines/growth factors could exert their anticancer ability in synergy with the cell-to-cell-interaction, as demonstrated by the close cell interference/interaction observed in the in vitro co-culture model (Figure 2).

On the other hand, the in vivo treatment of tumors with MSCs that secrete VEGF should promote angiogenesis in the xenograft and induce tumor growth. However, as reported by Otsu et al. [43], MSCs, locally injected into tumor tissue, are cytotoxic to newly forming vessels. Therefore, in our model, we tested whether angiogenesis was affected by MSCs by evaluating the expression of vascular marker CD31 in the explanted tumor nodules. The immunohistochemical analysis did not indicate changes in CD31 expression, suggesting that VEGF was not important in inhibiting or stimulating angiogenesis into the tumor mass. Therefore, our findings indicated that tumor regression does not seem to be related to the effects on tumor vascularity.

According to Sage et al. [21], MSCs migrate to pleural mesothelioma tumors in vivo when delivered both intravenously and intrapleurally, but a significant reduction in tumor growth was observed only with intravenous treatment due to the higher number of MSCs capable of tumor engraftment.

As the aim of our study was to evaluate the in vivo effect of MSCs on MPM, we preliminary verified in vitro the ability of MSCs to integrate and interact with mesothelioma cells in a context of cell-to-cell interaction by co-culturing mesothelioma cancer cells with engineered MSCs expressing Green fluorescent protein (GFP). The results confirmed that MSCs were well integrated with cancer cells and that also, in the condition of cell-to-cell contact, MSCs exert a significant inhibitory action on MPM cell proliferation. Based on these data, we evaluated the therapeutic efficacy of MSCs in a model of subcutaneous xenograft of a human MPM in nude mice by a treatment in situ with MSCs that were able to incorporate into tumors. A significant inhibition of tumor growth was observed by MSCs treatment at a level comparable with that observed after systemic treatment with paclitaxel. To better analyze tumor nodule composition, Masson’s Trichrome staining of sections was employed to evaluate the percentage of tissue occupied by the neoplastic, fibrotic and necrotic component; the morphometric analysis clearly confirmed that the neoplastic tissue was significantly reduced in mice treated with MSCs. Most relevant, the reduction in tumor growth was comparable among MSCs and paclitaxel-treated animals.

Taken together, our data support the hypothesis that in the MPM environment, the MSCs could have an important role in controlling tumor growth and this could open the way to consider MSCs as a possible therapeutic tool. In this regard, since MSCs have been shown to incorporate and then release different types of anticancer drugs (e.g., paclitaxel, gemcitabine, doxorubicin) [44,45], we suppose that MSCs could improve their basal anticancer efficacy once loaded with chemotherapy molecules. Whether this cell therapy approach could represent a new adjuvant therapy (also associated with the surgery) for human MPM remains to be investigated. However, the demonstration, for the first time, that MSCs per se can act as an “anti-tumor drug” against human MPM has a significant translational value that supports the possibility of future clinical trials with large-scale production in bioreactors, according to the GMP requested by the regulatory agencies.

## Data Availability

The data presented in this study are available on request from the corresponding author.

## Supplementary Materials

The following are available online at https://www.mdpi.com/article/10.3390/cells10061427/s1, Figure S1: Immunostaining of the MSTO-211H tumor xenograft sections with anti-mouse CD31 antibody, Figure S2: Immunostaining of the MSTO-211H-MSC tumor xenograft sections with anti-mouse CD31 antibody.

## References

[1] Carbone M., Ly B.H., Dodson R.F., Pagano I., Morris P.T., Dogan U.A., Gazdar A.F., Pass H.I., Yang H. (2012). Malignant mesothelioma: Facts, myths, and hypotheses. J. Cell Physiol..

[2] Peto J., Decarli A., La Vecchia C., Levi F., Negri E. (1999). The European mesothelioma epidemic. Br. J. Cancer.

[3] Fennell D.A., Gaudino G., O’Byrne K.J., Mutti L., van Meerbeeck J. (2008). Advances in the systemic therapy of malignant pleural mesothelioma. Nat. Clin. Pract. Oncol..

[4] Krug L.M., Pass H.I., Rusch V.W., Kindler H.L., Sugarbaker D.J., Rosenzweig K.E., Flores R., Friedberg J.S., Pisters K., Monberg M. (2009). Multicenter phase II trial of neoadjuvant pemetrexed plus cisplatin followed by extrapleural pneumonectomy and radiation for malignant pleural mesothelioma. J. Clin. Oncol..

[5] Mutti L., Peikert T., Robinson B.W.S., Scherpereel A., Tsao A.S., de Perrot M., Woodard G.A., Jablons D.M., Wiens J., Hirsch F.R. (2018). Scientific Advances and New Frontiers in Mesothelioma Therapeutics. J. Thorac. Oncol..

[6] Li W., Zhou Y., Yang J., Zhang X., Zhang H., Zhang T., Zhao S., Zheng P., Huo J., Wu H. (2015). Gastric cancer-derived mesenchymal stem cells prompt gastric cancer progression through secretion of interleukin-8. J. Exp. Clin. Cancer Res..

[7] Li G.C., Zhang H.W., Zhao Q.C., Sun L.I., Yang J.J., Hong L., Feng F., Cai L. (2016). Mesenchymal stem cells promote tumor angiogenesis via the action of transforming growth factor β1. Oncol. Lett..

[8] Houthuijzen J.M., Daenen L.G., Roodhart J.M., Voest E.E. (2012). The role of mesenchymal stem cells in anti-cancer drug resistance and tumour progression. Br. J. Cancer.

[9] Ramasamy R., Lam E.W., Soeiro I., Tisato V., Bonnet D., Dazzi F. (2007). Mesenchymal stem cells inhibit proliferation and apoptosis of tumor cells: Impact on in vivo tumor growth. Leukemia.

[10] Chanda D., Isayeva T., Kumar S., Hensel J.A., Sawant A., Ramaswamy G., Siegal G.P., Beatty M.S., Ponnazhagan S. (2009). Therapeutic potential of adult bone marrow-derived mesenchymal stem cells in prostate cancer bone metastasis. Clin. Cancer Res..

[11] Ohlsson L.B., Varas L., Kjellman C., Edvardsen K., Lindvall M. (2003). Mesenchymal progenitor cell-mediated inhibition of tumor growth in vivo and in vitro in gelatin matrix. Exp. Mol. Pathol..

[12] Ayuzawa R., Doi C., Rachakatla R.S., Pyle M.M., Maurya D.K., Troyer D., Tamura M. (2009). Naïve human umbilical cord matrix derived stem cells significantly attenuate growth of human breast cancer cells in vitro and in vivo. Cancer Lett..

[13] Yuan Z., Kolluri K.K., Gowers K.H., Janes S.M. (2017). TRAIL delivery by MSC-derived extracellular vesicles is an effective anticancer therapy. J. Extracell. Vesicles.

[14] Li L., Tian H., Chen Z., Yue W., Li S., Li W. (2011). Inhibition of lung cancer cell proliferation mediated by human mesenchymal stem cells. Acta Biochim. Biophys. Sin. (Shanghai).

[15] Ramdasi S., Sarang S., Viswanathan C. (2015). Potential of Mesenchymal Stem Cell based application in Cancer. Int. J. Hematol. Oncol. Stem Cell Res..

[16] Galland S., Stamenkovic I. (2020). Mesenchymal stromal cells in cancer: A review of their immunomodulatory functions and dual effects on tumor progression. J. Pathol..

[17] Hmadcha A., Martin-Montalvo A., Gauthier B.R., Soria B., Capilla-Gonzalez V. (2020). Therapeutic Potential of Mesenchymal Stem Cells for Cancer Therapy. Front. Bioeng. Biotechnol..

[18] Nwabo Kamdje A.H., Kamga P.T., Simo R.T., Vecchio L., Seke Etet P.F., Muller J.M., Bassi G., Lukong E., Goel R.K., Amvene J.M. (2017). Mesenchymal stromal cells’ role in tumor microenvironment: Involvement of signaling pathways. Cancer Biol. Med..

[19] Cortes-Dericks L., Froment L., Kocher G., Schmid R.A. (2016). Human lung-derived mesenchymal stem cell-conditioned medium exerts in vitro antitumor effects in malignant pleural mesothelioma cell lines. Stem Cell Res. Ther..

[20] Lathrop M.J., Sage E.K., Macura S.L., Brooks E.M., Cruz F., Bonenfant N.R., Sokocevic D., MacPherson M.B., Beuschel S.L., Dunaway C.W. (2015). Antitumor effects of TRAIL-expressing mesenchymal stromal cells in a mouse xenograft model of human mesothelioma. Cancer Gene Ther..

[21] Sage E.K., Kolluri K.K., McNulty K., Lourenco Sda S., Kalber T.L., Ordidge K.L., Davies D., Gary Lee Y.C., Giangreco A., Janes S.M. (2014). Systemic but not topical TRAIL-expressing mesenchymal stem cells reduce tumour growth in malignant mesothelioma. Thorax.

[22] Lisini D., Nava S., Pogliani S., Avanzini M.A., Lenta E., Bedini G., Mantelli M., Pecciarini L., Croce S., Boncoraglio G. (2019). Adipose tissue-derived mesenchymal stromal cells for clinical application: An efficient isolation approach. Curr. Res. Transl. Med..

[23] Lisini D., Nava S., Frigerio S., Pogliani S., Maronati G., Marcianti A., Coccè V., Bondiolotti G., Cavicchini L., Paino F. (2020). Automated Large-Scale Production of Paclitaxel Loaded Mesenchymal Stromal Cells for Cell Therapy Applications. Pharmaceutics.

[24] Petrella F., Coccè V., Masia C., Milani M., Salè E.O., Alessandri G., Parati E., Sisto F., Pentimalli F., Brini A.T. (2017). Paclitaxel-releasing mesenchymal stromal cells inhibit in vitro proliferation of human mesothelioma cells. Biomed. Pharmacother..

[25] Mossman T. (1983). Rapid colorimetric assay for cellular growth and survival: Application to proliferation and cytotoxicity assays. J. Immunol. Methods.

[26] Fumarola C., La Monica S., Alfieri R.R., Borra E., Guidotti G.G. (2005). Cell size reduction induced by inhibition of the mTOR/S6K-signaling pathway protects Jurkat cells from apoptosis. Cell Death Differ..

[27] Balducci L., Blasi A., Saldarelli M., Soleti A., Pessina A., Bonomi A., Coccè V., Dossena M., Tosetti V., Ceserani V. (2014). Immortalization of human adipose-derived stromal cells: Production of cell lines with high growth rate, mesenchymal marker expression and capability to secrete high levels of angiogenic factors. Stem Cell Res. Ther..

[28] Coccè V., Farronato D., Brini A.T., Masia C., Giannì A.B., Piovani G., Sisto F., Alessandri G., Angiero F., Pessina A. (2017). Drug Loaded Gingival Mesenchymal Stromal Cells (GinPa-MSCs) Inhibit In Vitro Proliferation of Oral Squamous Cell Carcinoma. Sci. Rep..

[29] Coccè V., Balducci L., Falchetti M.L., Pascucci L., Ciusani E., Brini A.T., Sisto F., Piovani G., Alessandri G., Parati E. (2017). Fluorescent Immortalized Human Adipose Derived Stromal Cells (hASCs-TS/GFP+) for Studying Cell Drug Delivery Mediated by Microvesicles. Anticancer Agents Med. Chem..

[30] Berenzi A., Steimberg N., Boniotti J., Mazzoleni G. (2015). MRT letter: 3D culture of isolated cells: A fast and efficient method for optimizing their histochemical and immunocytochemical analyses. Microsc. Res. Tech..

[31] La Monica S., Cretella D., Bonelli M., Fumarola C., Cavazzoni A., Digiacomo G., Flammini L., Barocelli E., Minari R., Naldi N. (2017). Trastuzumab emtansine delays and overcomes resistance to the third-generation EGFR-TKI osimertinib in NSCLC EGFR mutated cell lines. J. Exp. Clin. Cancer Res..

[32] La Monica S., Minari R., Cretella D., Flammini L., Fumarola C., Bonelli M., Cavazzoni A., Digiacomo G., Galetti M., Madeddu D. (2019). Third generation EGFR inhibitor osimertinib combined with pemetrexed or cisplatin exerts long-lasting anti-tumor effect in EGFR-mutated pre-clinical models of NSCLC. J. Exp. Clin. Cancer Res..

[33] Christodoulou I., Goulielmaki M., Devetzi M., Panagiotidis M., Koliakos G., Zoumpourlis V. (2018). Mesenchymal stem cells in preclinical cancer cytotherapy: A systematic review. Stem Cell Res. Ther..

[34] Park C.W., Kim K.S., Bae S., Son H.K., Myung P.K., Hong H.J., Kim H. (2009). Cytokine secretion profiling of human mesenchymal stem cells by antibody array. Int. J. Stem Cells.

[35] Wu Y., Hoogduijn M.J., Baan C.C., Korevaar S.S., de Kuiper R., Yan L., Wang L., van Besouw N.M. (2017). Adipose Tissue-Derived Mesenchymal Stem Cells Have a Heterogenic Cytokine Secretion Profile. Stem Cells Int..

[36] Abdul Rahim S.N., Ho G.Y., Coward J.I. (2015). The role of interleukin-6 in malignant mesothelioma. Transl. Lung Cancer Res..

[37] Assoni A., Coatti G., Valadares M.C., Beccari M., Gomes J., Pelatti M., Mitne-Neto M., Carvalho V.M., Zatz M. (2017). Different Donors Mesenchymal Stromal Cells Secretomes Reveal Heterogeneous Profile of Relevance for Therapeutic Use. Stem Cells Dev..

[38] Shrihari T.G. (2017). Dual role of inflammatory mediators in cancer. Ecancermedicalscience.

[39] Lee M.W., Ryu S., Kim D.S., Lee J.W., Sung K.W., Koo H.H., Yoo K.H. (2019). Mesenchymal stem cells in suppression or progression of hematologic malignancy: Current status and challenges. Leukemia.

[40] Pokrovskaya L.A., Zubareva E.V., Nadezhdin S.V., Lysenko A.S., Litovkina T.L. (2020). Biological activity of mesenchymal stem cells secretome as a basis for cell-free therapeutic approach. Res. Results Pharmacol..

[41] Mirabdollahi M., Haghjooyjavanmard S., Sadeghi-Aliabadi H. (2019). An anticancer effect of umbilical cord-derived mesenchymal stem cell secretome on the breast cancer cell line. Cell Tissue Bank.

[42] Leuning D.G., Beijer N.R.M., du Fossé N.A., Vermeulen S., Lievers E., van Kooten C., Rabelink T.J., Boer J. (2018). The cytokine secretion profile of mesenchymal stromal cells is determined by surface structure of the microenvironment. Sci. Rep..

[43] Otsu K., Das S., Houser S.D., Quadri S.K., Bhattacharya S., Bhattacharya J. (2009). Concentration-dependent inhibition of angiogenesis by mesenchymal stem cells. Blood.

[44] Pessina A., Bonomi A., Coccè V., Invernici G., Navone S., Cavicchini L., Sisto F., Ferrari M., Viganò L., Locatelli A. (2011). Mesenchymal stromal cells primed with paclitaxel provide a new approach for cancer therapy. PLoS ONE.

[45] Bonomi A., Sordi V., Dugnani E., Ceserani V., Dossena M., Coccè V., Cavicchini L., Ciusani E., Bondiolotti G., Piovani G. (2015). Gemcitabine-releasing mesenchymal stromal cells inhibit in vitro proliferation of human pancreatic carcinoma cells. Cytotherapy.

