# Supplementary material for: Inhibition of Human Malignant Pleural Mesothelioma Growth by Mesenchymal Stromal Cells
Source: Cells. 2021 Jun 8;10(6):1427. doi: 10.3390/cells10061427 (PMC8227879; doi:10.3390/cells10061427)
Supplement: Supplementary file 1 [file cells-10-01427-s001.zip › cells-1179298-supplementary.pdf]

Supplementary Materials

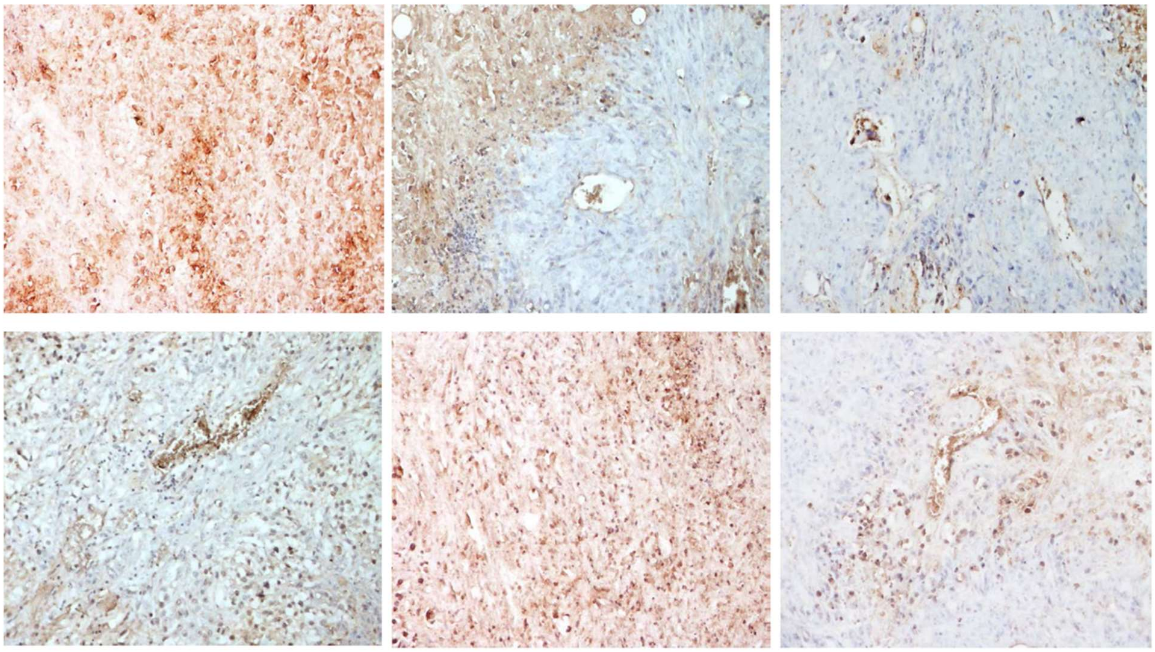

Figure S1. Immunostaining of the MSTO-211H tumor xenograft sections with anti-mouse CD31 antibody. Different microscopic fields (100× magnification) of MSTO-211H xenografts are shown. The pictures reported are representative of the analysis of 30 different microscopic fields.

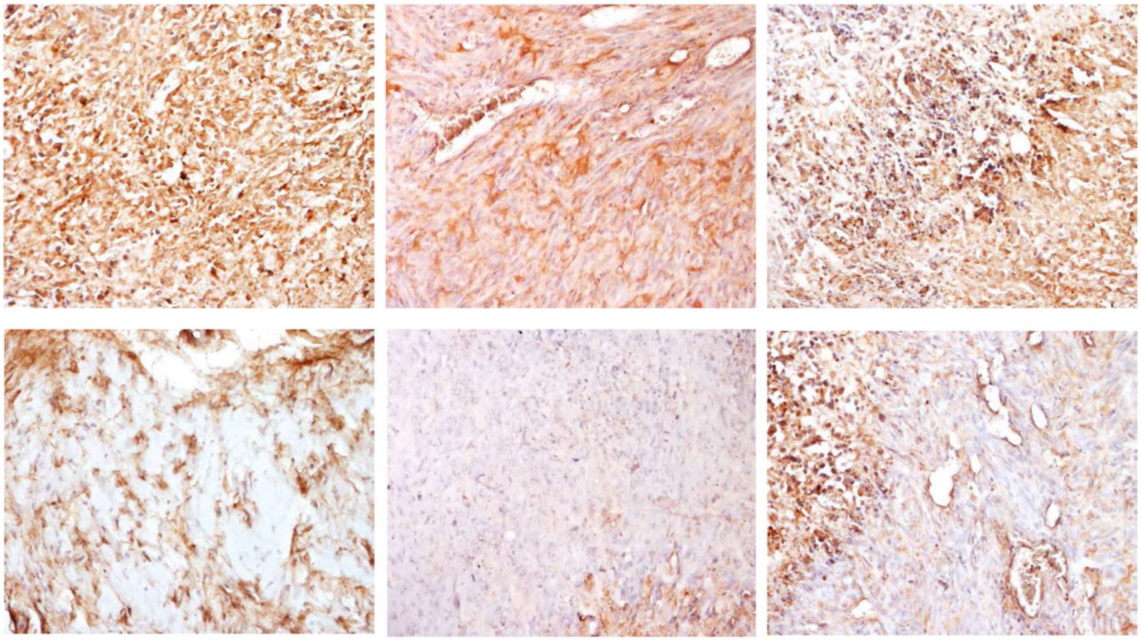

Figure S2. Immunostaining of the MSTO-211H-MSC tumor xenograft sections with anti-mouse CD31 antibody. Different microscopic fields (100× magnification) of MSTO-211H-MSC xenografts are shown. The pictures reported are representative of the analysis of 30 different microscopic fields.
